# Integrative co-registration of elemental imaging and histopathology for enhanced spatial multimodal analysis of tissue sections through TRACE

**DOI:** 10.1093/bioadv/vbaf001

**Published:** 2025-01-07

**Authors:** Yunrui Lu, Serin Han, Aruesha Srivastava, Neha Shaik, Matthew Chan, Alos Diallo, Naina Kumar, Nishita Paruchuri, Hrishikesh Deosthali, Vismay Ravikumar, Kevin Cornell, Elijah Stommel, Tracy Punshon, Brian Jackson, Fred Kolling, Linda Vahdat, Louis Vaickus, Jonathan Marotti, Sunita Ho, Joshua Levy

**Affiliations:** Department of Pathology and Laboratory Medicine, Dartmouth Health, Lebanon, NH 03766, United States; Dartmouth College, Geisel School of Medicine, Hanover, NH 03766, United States; Department of Pathology and Laboratory Medicine, Dartmouth Health, Lebanon, NH 03766, United States; California Institute of Technology, Pasadena, CA 91125, United States; Cupertino High School, Cupertino, CA 95014, United States; Dartmouth College, Geisel School of Medicine, Hanover, NH 03766, United States; Department of Pathology and Laboratory Medicine, Dartmouth Health, Lebanon, NH 03766, United States; Langley High School, McLean, VA 22101, United States; Thomas Jefferson High School for Science and Technology, Alexandria, VA 22312, United States; Liberal Arts and Science Academy, Austin, TX 78721, United States; Andover High School, Andover, MA 01810, United States; Department of Pathology and Laboratory Medicine, Dartmouth Health, Lebanon, NH 03766, United States; Department of Neurology, Dartmouth Health, Lebanon, NH 03766, United States; Department of Pathology and Laboratory Medicine, Dartmouth Health, Lebanon, NH 03766, United States; Department of Neurology, Dartmouth Health, Lebanon, NH 03766, United States; Dartmouth College, Geisel School of Medicine, Hanover, NH 03766, United States; Department of Biological Sciences, Dartmouth College, Hanover, NH 03766, United States; Dartmouth College, Geisel School of Medicine, Hanover, NH 03766, United States; Department of Earth Sciences, Dartmouth College, Hanover, NH 03766, United States; Dartmouth College, Geisel School of Medicine, Hanover, NH 03766, United States; Dartmouth Cancer Center, Lebanon, NH 03766, United States; Dartmouth Cancer Center, Lebanon, NH 03766, United States; Department of Medicine, Dartmouth Health, Lebanon, NH 03766, United States; Department of Pathology and Laboratory Medicine, Dartmouth Health, Lebanon, NH 03766, United States; Department of Pathology and Laboratory Medicine, Dartmouth Health, Lebanon, NH 03766, United States; School of Dentistry, University of California San Francisco, San Francisco, CA 94143, United States; Department of Pathology and Laboratory Medicine, , Cedars Sinai Medical Center, Los Angeles, CA 90048, United States; Department of Computational Biomedicine, Cedars Sinai Medical Center, Los Angeles, CA 90048, United States

## Abstract

**Summary:**

Elemental imaging provides detailed profiling of metal bioaccumulation, offering more precision than bulk analysis by targeting specific tissue areas. However, accurately identifying comparable tissue regions from elemental maps is challenging, requiring the integration of hematoxylin and eosin (H&E) slides for effective comparison. Facilitating the streamlined co-registration of whole slide images (WSI) and elemental maps, TRACE enhances the analysis of tissue regions and elemental abundance in various pathological conditions. Through an interactive containerized web application, TRACE features real-time annotation editing, advanced statistical tools, and data export, supporting comprehensive spatial analysis. Notably, it allows for comparison of elemental abundances across annotated tissue structures and enables integration with other spatial data types through WSI co-registration.

**Availability and implementation:**

Available on the following platforms—GitHub: *jlevy44/trace_app*, PyPI: *trace_app*, Docker: *joshualevy44/trace_app*, Singularity: docker://*joshualevy44/trace_app*.

## 1 Implementation

Finding where metals are situated in tissues is a new and challenging area, similar to the task of locating genes within tissues, which is vital for unraveling the complexities of various biological systems (see Supplementary Materials, section “Importance of Studying Metal Bioaccumulation”). Traditional measurements of elemental abundance, on a bulk scale, tend to neglect the intricacies and disruptions of metal homeostasis within specific tissue architectures, obscuring critical associations and insights (Moses and Pachter 2022). Spatially resolved metal analysis through techniques like laser ablation inductively coupled plasma time-of-flight mass spectrometry (LA-ICPTOF-MS) offers detailed maps of multi-elemental distributions at one-micron resolution (Bussweiler *et al.* 2017, Löhr *et al.* 2019, Theiner *et al.* 2019, Clases and Gonzalez de Vega 2022a,b, Chang *et al.* 2022, da Silva and Arruda 2023). Resulting biomolecular changes governing metal transport or biological pathways and cell-types disrupted by local metal deposits may be identified with immunohistochemistry (IHC), imaging mass cytometry (IMC), or multiplex immunofluorescence (mIF) (Niedzwiecki *et al.* 2016). Identifying where metals and their biological correlates occur within tissue typically involves pathologist annotations of relevant tissue structures on hematoxylin and eosin (H&E) or IHC slides. Alternatively, this process can be automated through the use of computer vision technologies, such as deep learning algorithms, which excel at autonomously annotating highly complex tissue regions and identifying cellular phenotypes with minimal human oversight (LeCun *et al.* 2015, Reddy *et al.* 2022).

Spatial multimodal co-registration workflows have been developed which align H&E slides (widely considered the gold standard) with IHC, IMC, mIF, and other spatial molecular modalities (e.g. spatial transcriptomics, ST) (Gatenbee *et al.* 2023). Such alignment offers numerous advantages for data preparation for integrative spatial multimodal analyses, leading to a more comprehensive understanding of spatial biomolecular heterogeneity and insights into multiple mechanistic pathways. Co-registration of spatial multimodal datasets can be automated (Heiser *et al.* 2023). However, this process often requires the placement of manual fiducials to facilitate alignment, especially when data have varying spatial scales (0.25 μm/pixel for H&E versus 1–10 μm/pixel), feature dimensionality (typically three color channels versus 100–200 elements), or custom-trimming of regions of interest. These differences typically complicate automated integration.

In light of these complexities, integrating histopathological and spatial molecular information for spatial elemental analysis through co-registration has been limited in the metallomics community due to the lack of user-friendly tools for such integration. Currently, the standard approach for tissue region labeling involves viewing annotated H&E images alongside elemental images and manually replicating the same annotations on the elemental images to facilitate further analysis (Paul *et al.* 2021). Because elemental imaging is a destructive process, this method requires profiling serial sections corresponding to the H&E images. This can potentially lead to misidentification of features and inaccuracies in labeling tissue within the elemental images.

Although co-registration of histopathological and elemental imaging modalities has been proposed within the elemental imaging community, these methods have not seen widespread adoption. A prior review of mass spectrometry imaging (MSI) co-registration methods highlighted several limitations: most tools are not open source, require image analysis expertise to implement custom workflows, and lack downstream analysis options that incorporate pathologist annotations (Balluff *et al.* 2022). Specifically, these tools do not support the transfer of annotated tissue regions from H&E sections to elemental images.

Co-registration methods generally rely on generating and comparing binary masks based on pixel-by-pixel locations of whole, intact tissue sections or on extracting multidimensional features for each pixel. However, these methods do not account for issues such as incomplete profiling of elements, regions with missing data, tissue sections that are scored or significantly distorted during processing, or spatial mismatches between imaging modalities. Additionally, the extraction of multidimensional pixel features may not align well with specific modalities and can be challenging for non-experts to interpret and troubleshoot. While integration of tissue autofluorescence can guide/improve co-registration, data preparation and analysis face similar challenges, especially for research groups without relevant expertise (Patterson *et al.* 2018).

Moreover, data standards like imzML, developed over a decade ago, are still employed in some co-registration tools (Römpp *et al.* 2011). Yet, these standards are not compatible with newly rasterized imaging formats required for downstream analysis with advanced computational methods and spatial multimodal data modalities (e.g. SpatialData) (Marconato *et al.* 2024).

**Figure 1.**
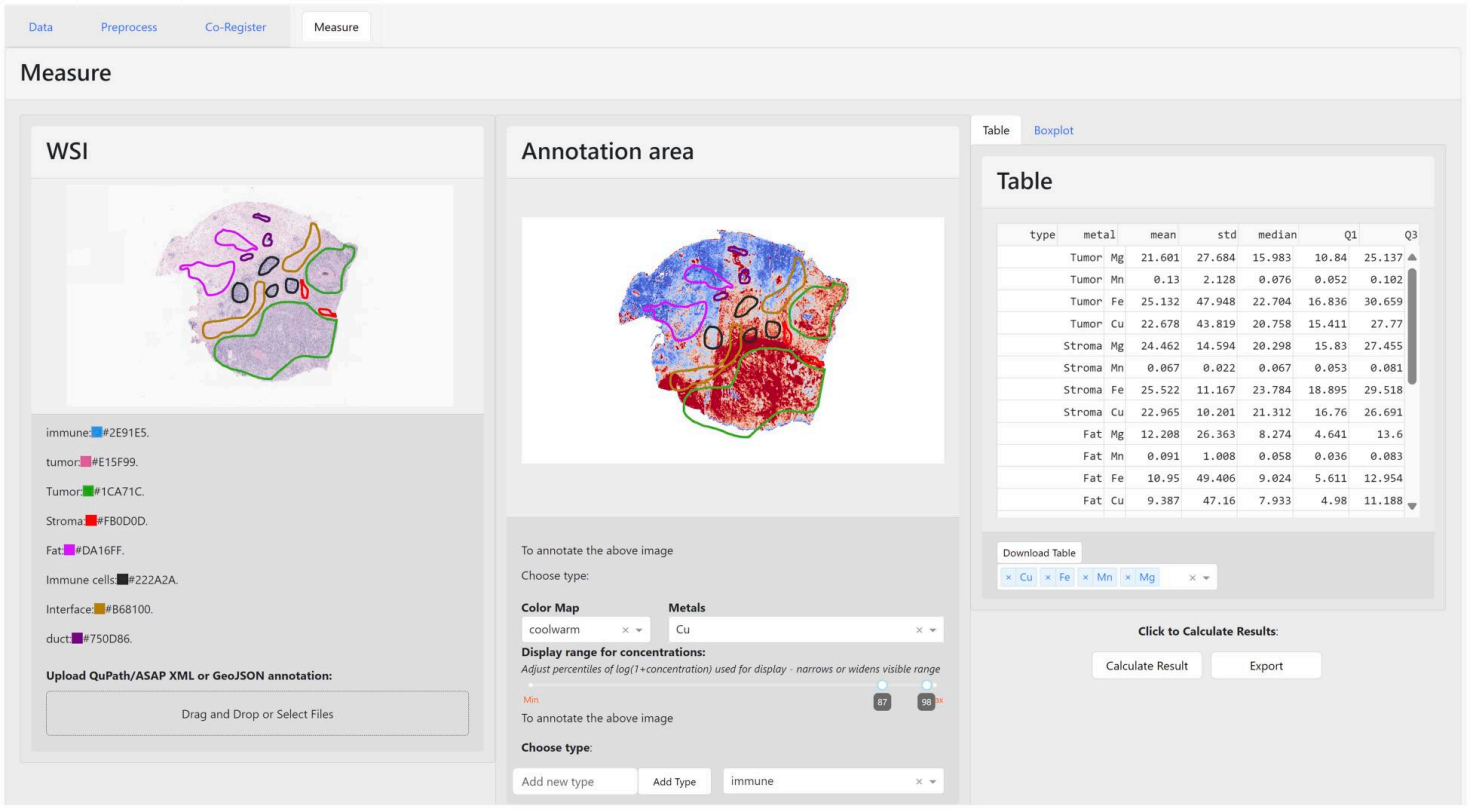
Transfer of pathologist annotations to LA-ICPMS elemental image through co-registration with TRACE.

In this article, we introduce TRACE—Tissue Region Analysis through Co-registration of Elemental Maps. Developed by the Biomedical National Elemental Imaging Resource (BNEIR), TRACE is an interactive web application that facilitates the co-registration of high-resolution whole slide images (WSI) with elemental maps, encompassing various imaging formats and a range of elemental imaging techniques (e.g. LA-ICPMS, XRF). TRACE enables comparisons of metal abundance across different, annotated tissue structures and data exported from this application also can be readily integrated with additional spatial genomics assays, such as spatial transcriptomics (Ståhl *et al.* 2016, Berglund *et al.* 2018, Stark *et al.* 2019, Ji *et al.* 2020, PALISI Pediatric Intensive Care Influenza (PICFLU) Investigators *et al.* 2020, Fawkner-Corbett *et al.* 2021, Garcia-Alonso *et al.* 2021, Maynard *et al.* 2021, Meylan *et al.* 2022). TRACE allows for a streamlined data integration, preprocessing, co-registration, and initial analysis comparing elemental abundance by tissue regions through the following collection of modules (Fig. 1, Supplementary Fig. S1):

1. **Efficient data management through data module:** This module simplifies the organization, upload, and management of various pathology images (including H&E, IHC, mIF) and annotations in JSON/XML format from tools like QuPath and ASAP (Bankhead *et al.* 2017, Humphries *et al.* 2021, Escobar Díaz Guerrero *et al.* 2022). It also handles metal images from techniques such as MALDI, LA-ICPTOF-MS, and XRF in multiple formats (Paton *et al.* 2011). The data module streamlines the integration of elemental imaging data, accommodating files exported from software like iolite (analyses software to generate quantitative spatial maps of elements in histologic sections, biopsies included). These files, typically a series of single channel elemental maps in Excel format, are consolidated into single files following the Bio-Format and FAIR (findable, accessible, interoperable, reusable) standards (e.g. OME-ZARR, SpatialData) for streamlined access and use (Moore *et al.* 2021).
2. **Preprocessing and co-registration modules:** Once multi-channel elemental images are generated, tissue is detected using a custom workflow which aggregates elemental abundance across selected channels. This process includes smoothing of pseudo-log-transformed data using a Gaussian filter, followed by user-defined or custom thresholding and morphological operations to refine the image, such as removing extraneous objects and filling in gaps within contiguous areas. After preprocessing, co-registration takes place. While there are advanced co-registration workflows utilizing feature matching and nonlinear transformations between H&E and elemental maps, our approach is currently landmark-based. This allows for real-time labeling of similar histopathological regions of interest, enabling linear co-registration to merge H&E images with metal maps effectively. Customizable visualizations aid in enhancing landmark identification, which is vital for pathologists to correlate pathology findings with metal composition and to transfer annotated biomarkers or regions on WSI directly to elemental maps.
3. **Data visualization/analysis via the measure module:** This module facilitates the visualization of co-registered elemental maps alongside WSI. It provides advanced tools for precise annotation and measurement within specific regions. Users can upload, import, and synchronize annotations from WSI in various formats, including JSON, XML, or GeoJSON, which are compatible with QuPath/ASAP pathology annotation tools. TRACE enables real-time synchronization between H&E and metal maps images, enhancing data interpretation, and currently provides basic statistical analysis functionalities. Additionally, data labeled with this tool can be exported in standard bioimaging formats, such as OME-ZARR, facilitating further analysis of elemental data along with the associated annotations transferred from WSI.

TRACE was implemented using a Python Dash/Flask front end (Dabbas 2021), Python v3.9.

## 2 Results

TRACE was initially tested in five illustrative use cases, demonstrating broad applicability, with plans for more comprehensive, in-depth analyses for expanded cohorts in future works:

1. **Annotation transfer in breast cancer study**: Applied to breast tumors and normal adjacent tissue with varying HER2/HR/TNBC molecular subtypes and histologies, TRACE enabled the transfer of pathology annotations from WSI to examine architectural differences in elemental maps. The following regions were annotated by a pathologist using the QuPath annotation software: Duct, Fat, Immune Cells, Interface, Lobule, Normal Fibrous Stroma, Stroma, and Tumor. Architectures were compared if at least tumor and normal adjacent tissue were profiled as means of comparison. Preliminary results were derived using Bayesian hierarchical hurdle gamma regression models (Bürkner 2017, 2018, Carpenter *et al.* 2017), reporting differences in tissue architectures by tissue type (tumor/normal adjacent) and architecture (Supplementary Fig. S2, Supplementary Table S1), accounting for patient using random effects. It should be noted due to limited sample size that the returned results provide an example of what can be done and is only proof-of-concept and do not draw any conclusions reserved for more expansive study.
2. **Colorectal cancer case study with spatial transcriptomics**: As a proof-of-concept, TRACE was applied to a pT3 stage colorectal cancer case, integrating with spatial transcriptomics data (Fatemi *et al.* 2023, 2024). The 10x Genomics Visium CytAssist spatial transcriptomics (ST) assay (Janesick *et al.* 2023), capturing spatial gene expression variations within 55-µm spots, was aligned with 40X H&E-stained WSI (Leica Aperio GT450). Regions in and around the tumor were annotated by pathologists. ST data were clustered using UMAP embeddings and Leiden clustering and visualized, with further analysis left for a future work (Supplementary Fig. S3) (McInnes *et al.* 2017, 2018). Elemental imaging at 5-µm resolution utilized laser ablation inductively coupled plasma time-of-flight mass spectrometry (LA-ICPTOF-MS), on tissue sectioned from formalin-fixed, paraffin-embedded blocks. Prior to further analysis, co-registered data were exported from TRACE.
3. **Spatial transcriptomics breast cohort:** Following the same workflow as in the Colorectal Cancer Case Study, TRACE was applied to deparaffinized tissue from two Triple Negative Breast Cancer (TNBC) patients, profiling ST data and elemental imaging on serial sections. Corresponding spatial molecular data were co-registered and exported from TRACE for further analysis (Supplementary Fig. S4).
4. **Co-registration of mIF in kidney papillae:** This example demonstrates the integration of spatial proteomics with elemental maps by combining two co-registration workflows. A kidney papillae block was sectioned into five serial tissue sections: the first was stained with H&E and excluded from the analysis, while four intermediate sections were analysed using mIF, incorporating 2–3 stains per section. Each section featured specific markers with DAPI for nuclei segmentation. The final section was analysed using LA-ICP-MS. All mIF images were co-registered using VALIS to create a composite mIF image containing all markers, featuring a six-plex protein panel (DAPI, PIEZO1, HIF-1α/Zn, MFN2, TRPV4). The composite mIF data were aligned with elemental maps using TRACE to facilitate integration of single-cell protein expression data extracted using a neural network through usage of the same transformation parameters (Supplementary Fig. S5, Supplementary Table S2).
5. **Central nervous system tissue:** In this case, serial H&E and elemental images of neocortex tissue from a single patient were taken and co-registered. This alignment revealed localized metal concentrations associated with specific tissue structures (Supplementary Fig. S6).

## 3 Benefits and future direction

TRACE permits flexible integration of histological and spatial transcriptomic data with elemental imaging analysis. This tool is available via PyPI (*trace-app*), GitHub (*jlevy44/trace_app*), Docker (*joshualevy44/trace_app*), and Singularity (docker://*joshualevy44/trace_app*), making the tool reproducible, accessible/sharable, and operating system agnostic. A video tutorial illustrating usage of the tool and a user-friendly application launcher (executable file) can be found in the GitHub repository. Applying this web application across various tissue types will broaden the scope and validity of our research in identifying prognostic elemental and transcriptomic markers within specific tissue structures. The current version allows for the co-registration of WSI with elemental maps. Future updates include a second release, which will integrate multiple modalities into a single multimodal data array. This approach will enable the integration of multiplexed spatial imaging and genomics assays with elemental imaging and histology. Such integration facilitates metals-based pathway analysis at a resolution approaching that of single cells. However, it is important to recognize that elemental imaging is generally a destructive process, necessitating the analysis to be conducted on consecutive tissue sections. Linking individual cell profiles with elemental abundance poses significant challenges, often requiring studies at a broader scale, such as examining the relationship between cellular interaction densities and the presence of metals and their mixtures. Furthermore, dewaxing of formalin-fixed paraffin-embedded tissue sections is currently recommended for LA-ICPTOF-MS applications to reduce the likelihood of signal intensity fluctuations though the impact of paraffin removal on metal distribution is currently understudied. While further spatial genomics analysis can currently be achieved by exporting and analysing co-registered data, future enhancements will offer interactive clustering tools, identification of metal hotspots using local spatial autocorrelation statistics, advanced machine learning analytics (Paul *et al.* 2021), and enhanced nonlinear co-registration techniques. Although several multimodal analysis tools have been developed for this purpose, an in-depth discussion and comparison of these downstream tools falls outside the scope of this manuscript. Instead, this work focuses on data preparation for such analyses and the initial comparison of tissue regions based on elemental abundance. References have been provided for readers interested in exploring these tools further (Ji *et al.* 2020, Bredikhin *et al.* 2022, Velten *et al.* 2022, Park *et al.* 2022, Arutyunyan *et al.* 2023, Dimitrov *et al.* 2024, Marconato *et al.* 2024, Vicari *et al.* 2024). Deployment on cloud platforms like Amazon Web Services is planned to improve accessibility. Additionally, high-resolution viewing of highly multiplexed imaging will be enabled through bioimaging software like ViV, Vitessce, and Avivator, using standard NGFF formats (Keller *et al.* 2021, Manz *et al.* 2022). The integration of these features, which currently require external analysis of exported data, will further enhance the application’s capabilities.

## Supplementary Material

vbaf001_Supplementary_Data

## Data Availability

Access to manuscript data is limited due to patient privacy concerns. All data produced in the present study are available upon reasonable request. Requests should be directed to senior author Dr. Joshua Levy (email: joshua.levy@cshs.org).
