# Supplementary material for: Integrative co-registration of elemental imaging and histopathology for enhanced spatial multimodal analysis of tissue sections through TRACE
Source: Bioinform Adv. 2025 Jan 7;5(1):vbaf001. doi: 10.1093/bioadv/vbaf001 (PMC11742137; doi:10.1093/bioadv/vbaf001)
Supplement: vbaf001_Supplementary_Data [file vbaf001_supplementary_data.zip › e10ea_trace_suppl_materials_revisions_submit_accept.docx]

**Supplementary Materials**

**Importance of Studying Metal Bioaccumulation**

There are at least twenty known trace elements that are essential for human survival, relating to energy metabolism, enzymatic activity, maintaining osmotic pressure, transport proteins, amongst others (Jomova *et al.*, 2022). However, deviations from normal metal homeostasis, whether due to a deficiency or excess of these essential elements, or the introduction of toxic elements from environmental exposure, diet, or lifestyle, are linked to the development/progression of various health conditions (Mehri, 2020). For instance, in the context of cancer, elements like copper, cadmium, and iron play significant roles in mitochondrial metabolism, cell proliferation, tumorigenesis, and proangiogenic pathways (Liao *et al.*, 2020). As another example, villi in the placenta mediate the transfer of nutrients and contaminants between mother and fetus (Zhang *et al.*, 2015), with bioaccumulation reflecting disruptions in homeostasis (Punshon *et al.*, 2019).

Metal transporter genes are essential in maintaining homeostasis through the redistribution of elements, and their conservation across species underscores their significance as a key molecular mechanism. Understanding the disruptions in metal homeostasis could unveil novel biomarkers and therapeutic targets. For example, the competitive binding of metals to these transporters can affect the bioaccumulation of toxic metals, offering a protective effect (Brooks *et al.*, 2016). Additionally, copper depletion therapies have shown promise in hindering tumor migration, invasion, and metastasis (Liu *et al.*, 2021; Chan *et al.*, 2017; Ramchandani *et al.*, 2021; Akerfeldt *et al.*, 2017; Baldari *et al.*, 2019; Fatfat *et al.*, 2014; Gandin *et al.*, 2012; Lopez *et al.*, 2019; Denoyer *et al.*, 2015; Gupte and Mumper, 2009). This is hypothesized to occur through two primary mechanisms: firstly, by reducing tumor metabolism (Ge *et al.*, 2022; Finney *et al.*, 2009; Kozono *et al.*, 2018; Chellan and Sadler, 2015; Fouani *et al.*, 2017), and secondly, by limiting the remodeling of the collagen extracellular matrix, which is crucial for tumor movement (Naji *et al.*, 2019; Shi *et al.*, 2021; Liang *et al.*, 2021; Wu *et al.*, 2022). Furthermore, lifestyle modifications, including dietary changes and minimizing exposure, are crucial for prevention and management.

Metal ions are essential for the functioning of many biomolecules, including proteins. For instance, Zinc is an essential nutrient and absence of this element is tied to upregulation hypoxia inducible factor HIF-1α in response to oxidative stress (Choi *et al.*, 2018; Marreiro *et al.*, 2017).

**
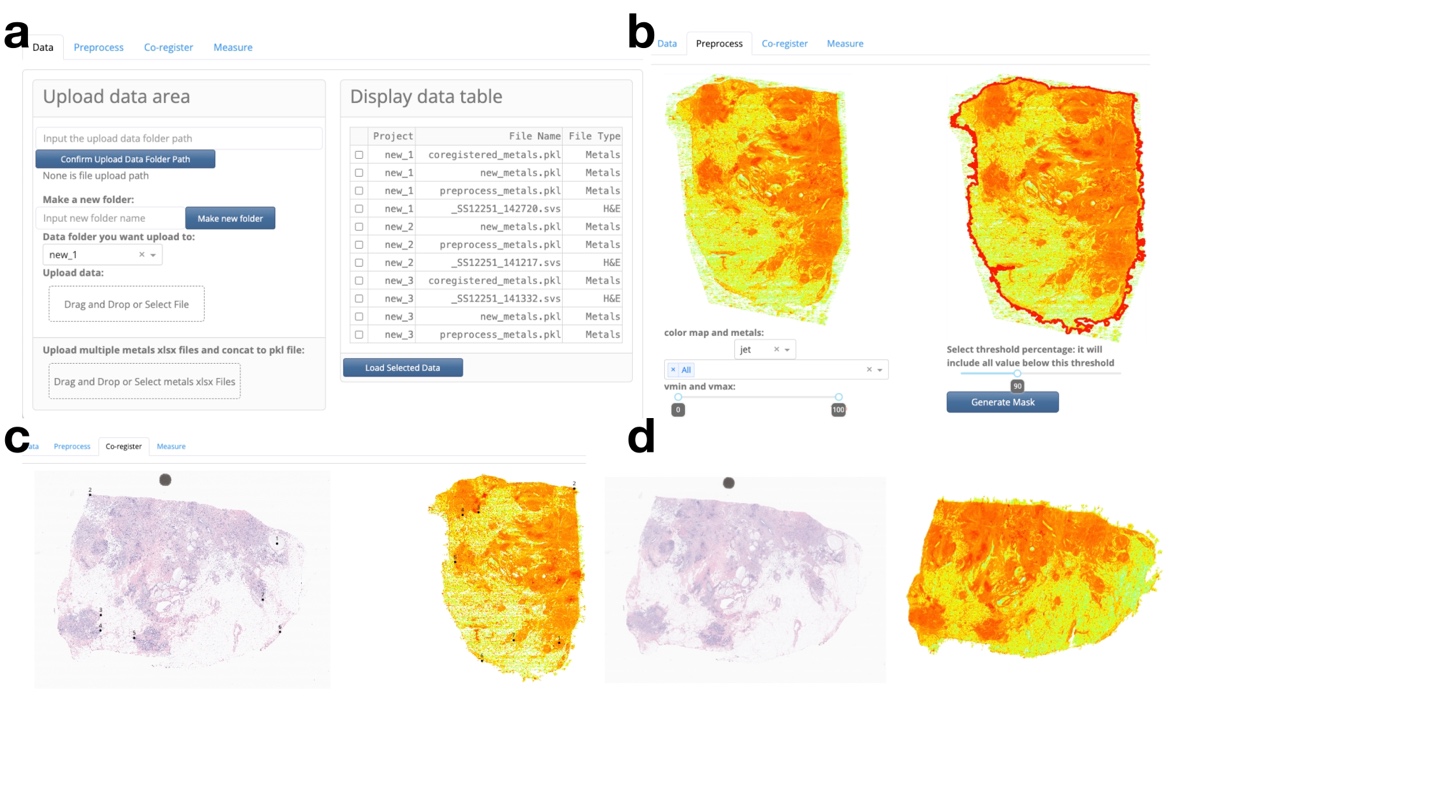
**

**Supplementary Figure 1: Demonstration of TRACE Web Application Components: a)** Data upload and management module selects H&E whole slide image and elemental map, **b)** Preprocessing module identifies tissue mask to remove background signal, **c)** Landmarks identified between H&E WSI and LA-ICPMS image to facilitate co-registration, **d)** Annotations are transferred from H&E section to co-registered elemental map for further measurement and downstream analysis

**
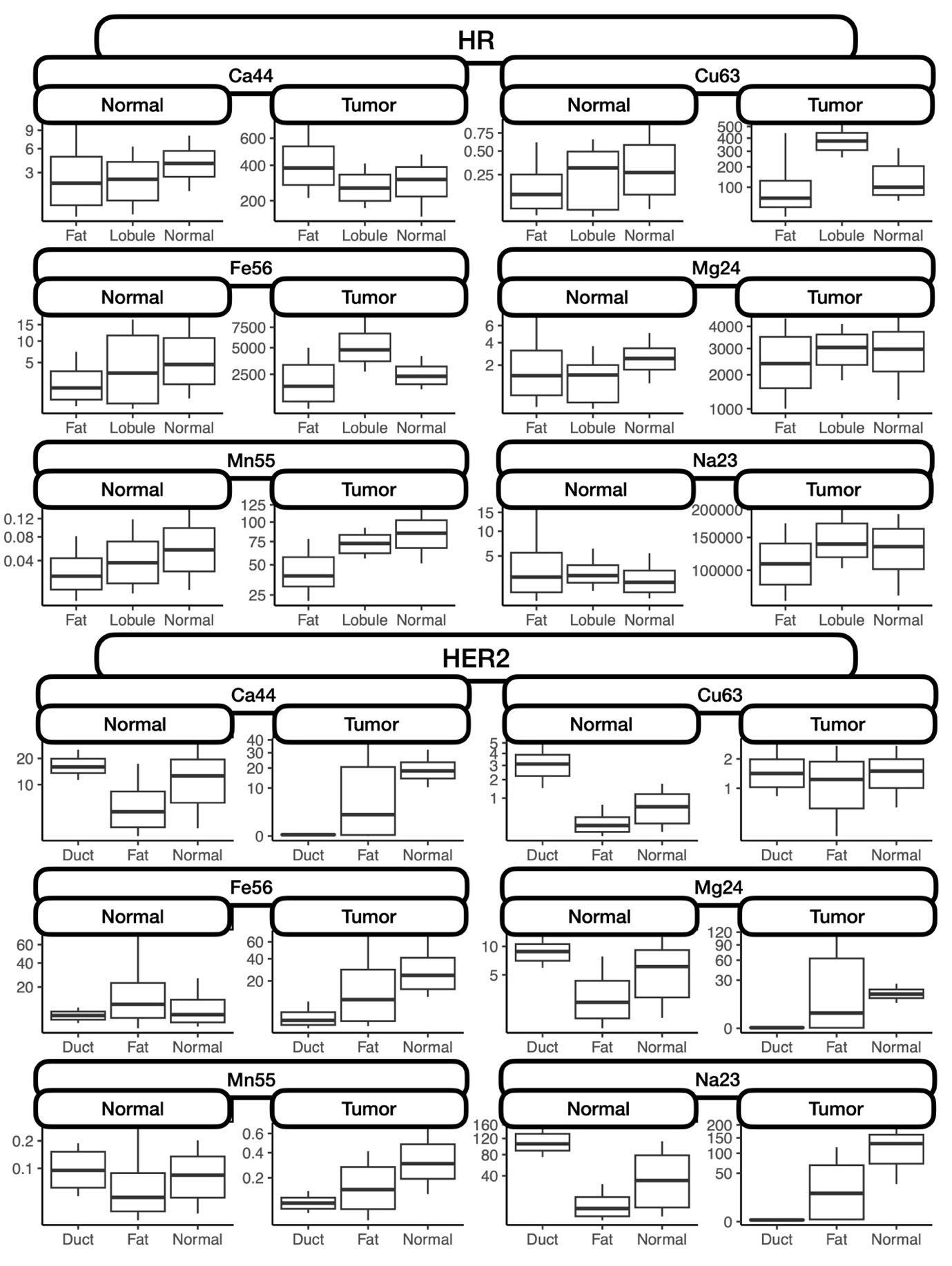
**

**Supplementary Figure 2: Boxplot comparison of elemental abundance in various tissue regions within select breast tumor sections, comparing ducts, fat and normal adjacent tissue**

**
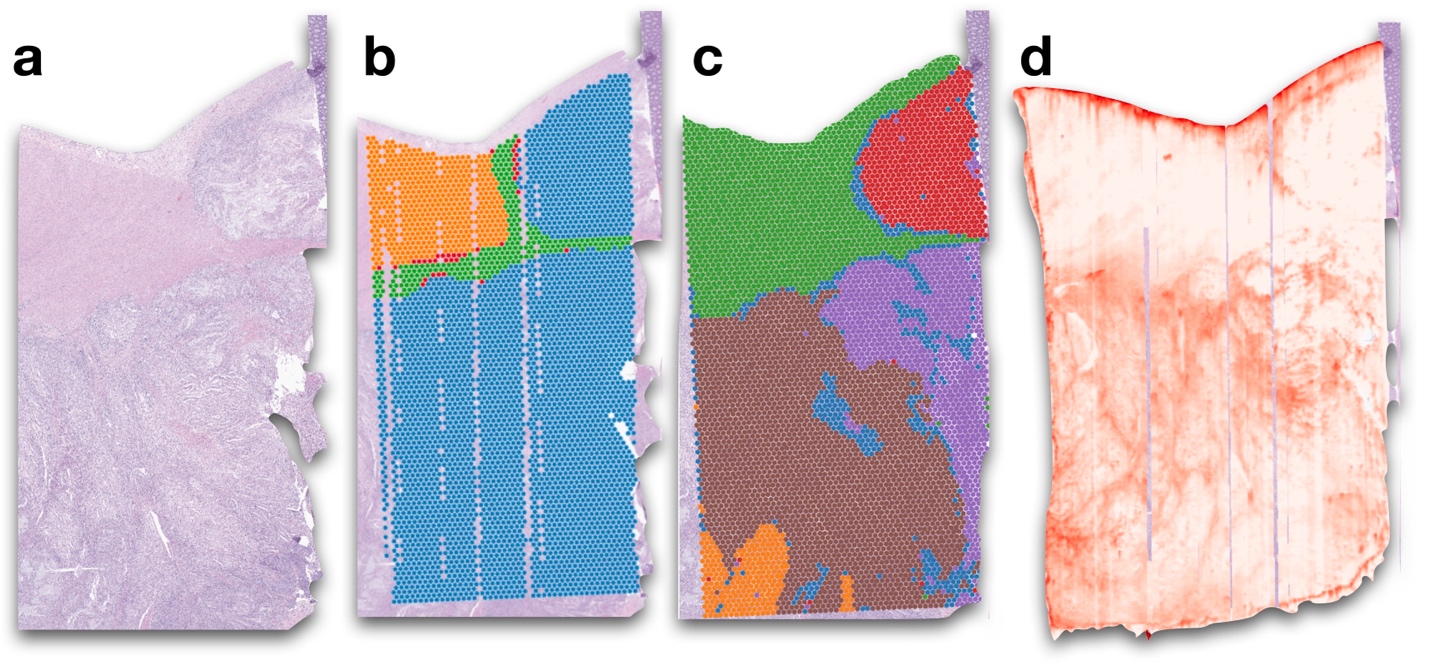
**

**Supplementary Figure 3: Co-Registration with TRACE on select Colorectal tumor section facilitates metals-based pathway assessment integrating: a)** H&E WSI, with **b)** pathologist annotations to delineate tissue architectures, **C)** spatial transcriptomics data, expression of nearly 18,000 genes at 55-micron resolution, leiden clustering performed based on similar expression profiles, and **D)** elemental imaging maps with LA-ICPTOF-MS at 5-micron resolution for all elements of the periodic table; image represents Fe concentration


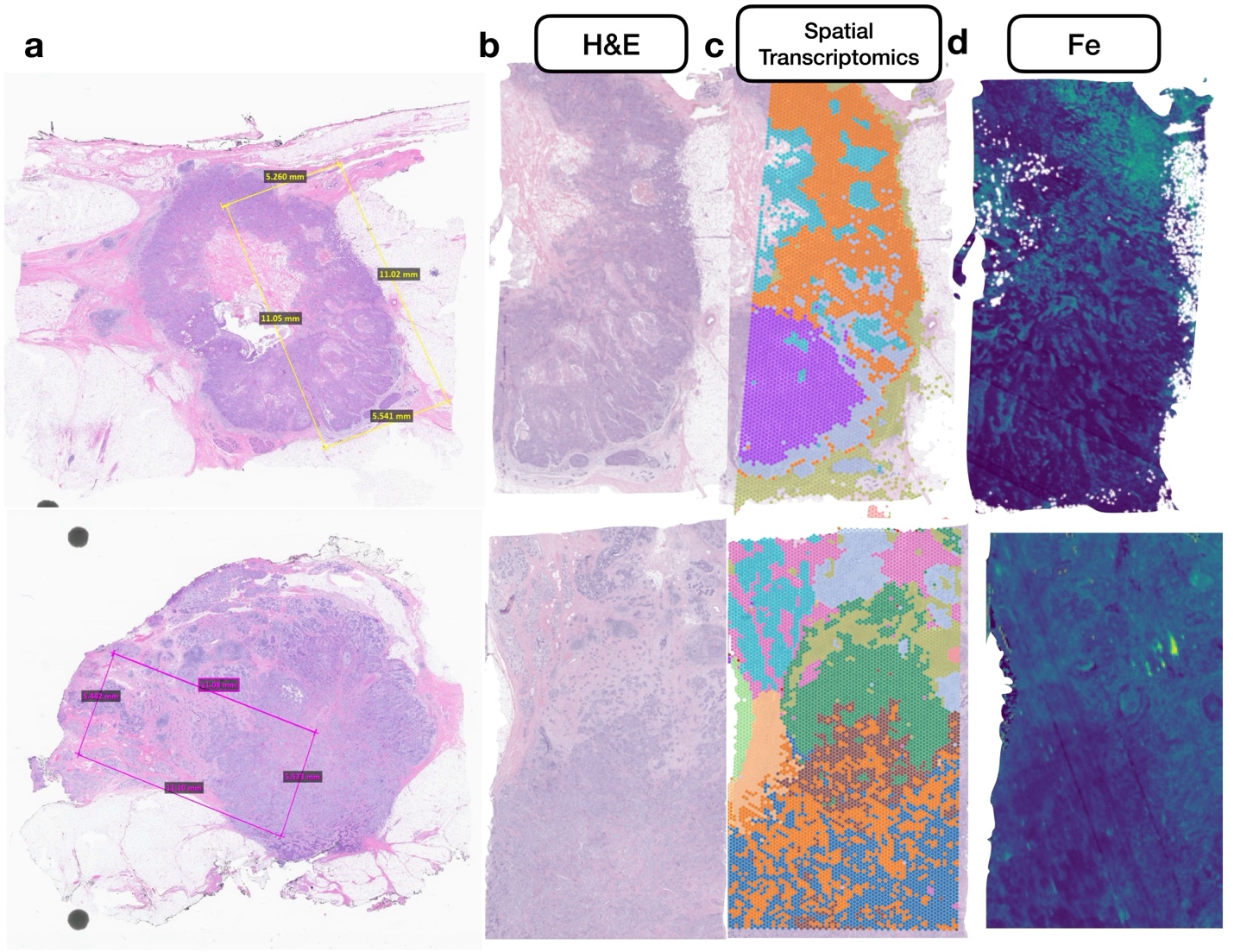


**Supplementary Figure 4: Co-Registration with TRACE on two select TNBC tumor sections facilitates metals-based pathway assessment integrating: a)** H&E WSI representing tissue region that was scored for further spatial transcriptomic analysis on a serial section, **b)** serial section H&E WSI where spatial transcriptomics profiling was done, **C)** spatial transcriptomics data, expression of nearly 18,000 genes at 55-micron resolution, leiden clustered based on similar expression profiles, and **D)** elemental imaging maps with LA-ICPTOF-MS at 5-micron resolution for all elements of the periodic table; image represents Fe concentration

**
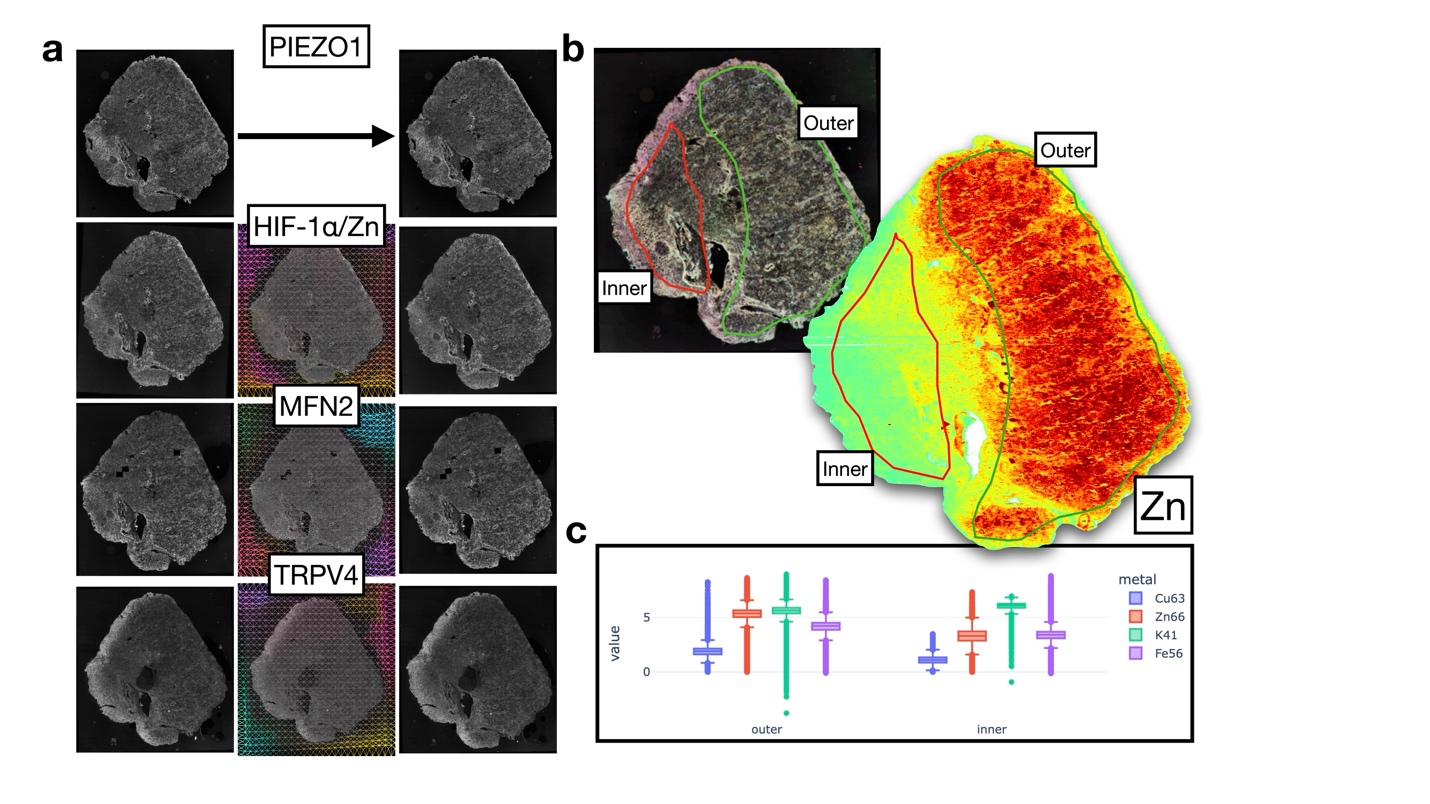
**

**Supplementary Figure 5:** **Demonstration of TRACE Co-Registration of LA-ICPTOF-MS to VALIS Constructed 6-plex mIF (DAPI, PIEZO1, HIF-1α/Zn, MFN2, TRPV4) for Example Kidney Papilla: a)** HIF-1α/Zn/DAPI, MFN2/DAPI, TRPV4/DAPI mIF images co-registered to PIEZO/DAPI mIF using VALIS software, **b)** Resulting mIF image is annotated using QuPath and co-registered to LA-ICPTOF-MS using TRACE for inner and outer zones in papilla, **c)** Screenshot of boxplot output of log-transformed elemental concentrations, comparing tissue regions. Selected tissue regions do not reflect typical histopathologic denominations and are used for demonstration purposes.

**
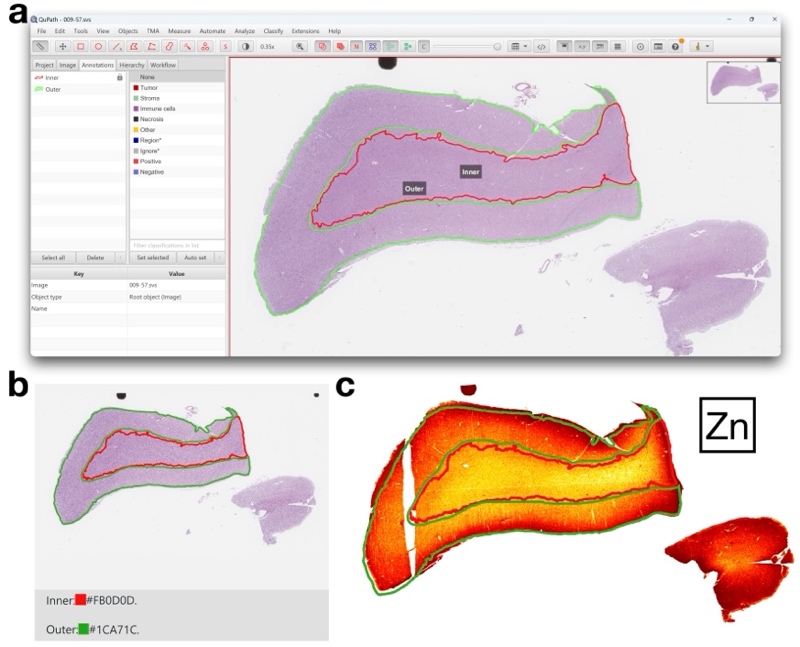
**

**Supplementary Figure 6: Demonstration of TRACE Co-Registration of Neocortex Tissue: a)** QuPath is used to annotate inner and outer motor cotex tissue regions, **b-c)** screenshots of TRACE web application after co-registration of **b)** H&E with **c)** Cu, Fe, Zn, Mn, Mg metal maps. Selected tissue regions do not reflect typical histopathologic denominations and are used for demonstration purposes.

**Supplementary Table 1: Statistical Results for LA-ICPMS differential elemental abundance between various tissue architectures for select specimens reflecting breast tumor subtypes**

| Subtype | Element | Parameter | Coefficient | 2.5% | 97.5% | p-value |
| --- | --- | --- | --- | --- | --- | --- |
| HER2 | **Ca44** | Duct > Fat, N | 0.22 | 0.19 | 0.26 | <0.001 |
|  |  | Duct > Normal Fibrous Stroma, N | 0.08 | 0.04 | 0.12 | <0.001 |
|  |  | Fat > Normal Fibrous Stroma, N | -0.14 | -0.20 | -0.09 | <0.001 |
|  |  | Duct > Fat, T | -0.42 | -0.47 | -0.38 | <0.001 |
|  |  | Duct > Normal Fibrous Stroma, T | -0.06 | -0.14 | 0.02 | 0.18 |
|  |  | Fat > Normal Fibrous Stroma, T | 0.37 | 0.27 | 0.46 | <0.001 |
|  | **Cu63** | Duct > Fat, N | 2.16 | 2.13 | 2.19 | <0.001 |
|  |  | Duct > Normal Fibrous Stroma, N | 1.55 | 1.52 | 1.59 | <0.001 |
|  |  | Fat > Normal Fibrous Stroma, N | -0.61 | -0.65 | -0.56 | <0.001 |
|  |  | Duct > Fat, T | 0.08 | 0.04 | 0.12 | <0.001 |
|  |  | Duct > Normal Fibrous Stroma, T | -0.53 | -0.60 | -0.46 | <0.001 |
|  |  | Fat > Normal Fibrous Stroma, T | -0.61 | -0.70 | -0.53 | <0.001 |
|  | **Fe56** | Duct > Fat, N | -1.90 | -1.95 | -1.84 | <0.001 |
|  |  | Duct > Normal Fibrous Stroma, N | -0.85 | -0.91 | -0.79 | <0.001 |
|  |  | Fat > Normal Fibrous Stroma, N | 1.05 | 0.97 | 1.13 | <0.001 |
|  |  | Duct > Fat, T | -1.39 | -1.46 | -1.32 | <0.001 |
|  |  | Duct > Normal Fibrous Stroma, T | -2.65 | -2.77 | -2.54 | <0.001 |
|  |  | Fat > Normal Fibrous Stroma, T | -1.26 | -1.41 | -1.12 | <0.001 |
|  | **Mg24** | Duct > Fat, N | -0.56 | -0.61 | -0.51 | <0.001 |
|  |  | Duct > Normal Fibrous Stroma, N | -1.11 | -1.16 | -1.06 | <0.001 |
|  |  | Fat > Normal Fibrous Stroma, N | -0.55 | -0.62 | -0.48 | <0.001 |
|  |  | Duct > Fat, T | -2.75 | -2.81 | -2.68 | <0.001 |
|  |  | Duct > Normal Fibrous Stroma, T | -1.60 | -1.71 | -1.50 | <0.001 |
|  |  | Fat > Normal Fibrous Stroma, T | 1.14 | 1.01 | 1.27 | <0.001 |
|  | **Mn55** | Duct > Fat, N | -1.42 | -1.47 | -1.37 | <0.001 |
|  |  | Duct > Normal Fibrous Stroma, N | -2.41 | -2.45 | -2.35 | <0.001 |
|  |  | Fat > Normal Fibrous Stroma, N | -0.99 | -1.06 | -0.92 | <0.001 |
|  |  | Duct > Fat, T | -0.42 | -0.48 | -0.36 | <0.001 |
|  |  | Duct > Normal Fibrous Stroma, T | -1.08 | -1.19 | -0.97 | <0.001 |
|  |  | Fat > Normal Fibrous Stroma, T | -0.66 | -0.79 | -0.53 | <0.001 |
|  | **Na23** | Duct > Fat, N | 0.90 | 0.87 | 0.93 | <0.001 |
|  |  | Duct > Normal Fibrous Stroma, N | 0.64 | 0.61 | 0.67 | <0.001 |
|  |  | Fat > Normal Fibrous Stroma, N | -0.26 | -0.30 | -0.21 | <0.001 |
|  |  | Duct > Fat, T | -0.24 | -0.28 | -0.20 | <0.001 |
|  |  | Duct > Normal Fibrous Stroma, T | 0.32 | 0.26 | 0.39 | <0.001 |
|  |  | Fat > Normal Fibrous Stroma, T | 0.57 | 0.48 | 0.64 | <0.001 |
| HR | **Ca44** | Fat > Lobule, N | 0.71 | 0.58 | 0.85 | <0.001 |
|  |  | Fat > Normal Fibrous Stroma, N | 0.21 | 0.17 | 0.25 | <0.001 |
|  |  | Lobule > Normal Fibrous Stroma, N | -0.51 | -0.65 | -0.36 | <0.001 |
|  |  | Fat > Lobule, T | 0.37 | 0.12 | 0.59 | 0.004 |
|  |  | Fat > Normal Fibrous Stroma, T | 0.14 | 0.02 | 0.26 | 0.022 |
|  |  | Lobule > Normal Fibrous Stroma, T | -0.22 | -0.48 | 0.05 | 0.094 |
|  | **Cu63** | Fat > Lobule, N | 0.15 | 0.03 | 0.25 | 0.008 |
|  |  | Fat > Normal Fibrous Stroma, N | -0.09 | -0.13 | -0.05 | <0.001 |
|  |  | Lobule > Normal Fibrous Stroma, N | -0.24 | -0.36 | -0.11 | <0.001 |
|  |  | Fat > Lobule, T | -0.60 | -0.80 | -0.42 | <0.001 |
|  |  | Fat > Normal Fibrous Stroma, T | 0.33 | 0.23 | 0.43 | <0.001 |
|  |  | Lobule > Normal Fibrous Stroma, T | 0.93 | 0.73 | 1.16 | <0.001 |
|  | **Fe56** | Fat > Lobule, N | -0.06 | -0.18 | 0.06 | 0.358 |
|  |  | Fat > Normal Fibrous Stroma, N | -0.22 | -0.25 | -0.18 | <0.001 |
|  |  | Lobule > Normal Fibrous Stroma, N | -0.16 | -0.28 | -0.03 | 0.012 |
|  |  | Fat > Lobule, T | -1.47 | -1.68 | -1.26 | <0.001 |
|  |  | Fat > Normal Fibrous Stroma, T | -0.56 | -0.65 | -0.47 | <0.001 |
|  |  | Lobule > Normal Fibrous Stroma, T | 0.91 | 0.68 | 1.15 | <0.001 |
|  | **Mg24** | Fat > Lobule, N | 0.36 | 0.24 | 0.47 | <0.001 |
|  |  | Fat > Normal Fibrous Stroma, N | -0.14 | -0.17 | -0.10 | <0.001 |
|  |  | Lobule > Normal Fibrous Stroma, N | -0.50 | -0.62 | -0.37 | <0.001 |
|  |  | Fat > Lobule, T | -1.16 | -1.37 | -0.96 | <0.001 |
|  |  | Fat > Normal Fibrous Stroma, T | -1.03 | -1.11 | -0.93 | <0.001 |
|  |  | Lobule > Normal Fibrous Stroma, T | 0.14 | -0.08 | 0.37 | 0.208 |
|  | **Mn55** | Fat > Lobule, N | 0.34 | 0.20 | 0.48 | <0.001 |
|  |  | Fat > Normal Fibrous Stroma, N | -1.47 | -1.51 | -1.42 | <0.001 |
|  |  | Lobule > Normal Fibrous Stroma, N | -1.81 | -1.96 | -1.66 | <0.001 |
|  |  | Fat > Lobule, T | 0.98 | 0.74 | 1.22 | <0.001 |
|  |  | Fat > Normal Fibrous Stroma, T | 0.74 | 0.59 | 0.89 | <0.001 |
|  |  | Lobule > Normal Fibrous Stroma, T | -0.24 | -0.51 | 0.05 | 0.088 |
|  | **Na23** | Fat > Lobule, N | -0.30 | -0.42 | -0.19 | <0.001 |
|  |  | Fat > Normal Fibrous Stroma, N | -0.24 | -0.28 | -0.21 | <0.001 |
|  |  | Lobule > Normal Fibrous Stroma, N | 0.06 | -0.06 | 0.19 | 0.344 |
|  |  | Fat > Lobule, T | -0.64 | -0.85 | -0.43 | <0.001 |
|  |  | Fat > Normal Fibrous Stroma, T | -0.41 | -0.51 | -0.31 | <0.001 |
|  |  | Lobule > Normal Fibrous Stroma, T | 0.23 | 0.00 | 0.47 | 0.054 |

**Supplementary Table 2: Direct Table Export from TRACE Web Application Comparing Elemental Concentrations at Outer and Inner Zones of Kidney Papilla Example,** Normalized to Reference Standard

| Tissue Region | Element | Mean | Std. Dev. | Median | Q1 | Q3 |
| --- | --- | --- | --- | --- | --- | --- |
| Outer | **Se80** | 5282.207 | 2573.68 | 5072.48 | 3607.093 | 6682.285 |
|  | **Na23** | 50935.012 | 19122.194 | 49610.025 | 38510.741 | 61641.313 |
|  | **Cu63** | 5.953 | 9.493 | 5.701 | 4.027 | 7.472 |
|  | **Zn66** | 223.334 | 109.874 | 214.102 | 151.652 | 282.872 |
|  | **Ca44** | 1189.449 | 1265.232 | 1088.531 | 854.732 | 1361.005 |
|  | **Mg24** | 550.704 | 464.81 | 511.122 | 313.647 | 716.838 |
|  | **K41** | 296.175 | 121.033 | 277.291 | 213.74 | 357.013 |
|  | **Co59** | 2.14 | 0.962 | 2.037 | 1.501 | 2.654 |
|  | **Fe56** | 69.841 | 35.411 | 67.537 | 46.555 | 89.489 |
|  | **As75** | 5.223 | 5.529 | 4 | 1.352 | 7.479 |
|  | **Mn55** | 1.735 | 4.796 | 1.526 | 0.804 | 2.335 |
|  | **All** | 53282.213 | 19437.722 | 52060.354 | 40936.131 | 64071.892 |
| Inner | **Se80** | 745.092 | 488.284 | 654.1 | 420.246 | 986.292 |
|  | **Na23** | 32411.104 | 14695.322 | 30864.433 | 21431.325 | 42247.329 |
|  | **Cu63** | 2.143 | 1.135 | 1.978 | 1.353 | 2.773 |
|  | **Zn66** | 30.15 | 20.64 | 26.17 | 16.604 | 40.048 |
|  | **Ca44** | 1364.545 | 1358.95 | 933.519 | 635.329 | 1736.521 |
|  | **Mg24** | 616.509 | 548.585 | 471.848 | 243.67 | 796.739 |
|  | **K41** | 440.051 | 124.681 | 437.402 | 356.526 | 521.764 |
|  | **Co59** | 1.899 | 0.847 | 1.831 | 1.345 | 2.378 |
|  | **Fe56** | 33.672 | 64.324 | 29.211 | 20.931 | 38.805 |
|  | **As75** | 2.725 | 3.019 | 2.178 | 0.957 | 4.779 |
|  | **Mn55** | -0.31 | 0.604 | -0.356 | -0.634 | -0.032 |
|  | **All** | 34902.716 | 14345.774 | 33200.006 | 24880.89 | 44130.31634 |

**References**

Akerfeldt,M.C. *et al.* (2017) Interactions of cisplatin and the copper transporter CTR1 in human colon cancer cells. *JBIC Journal of Biological Inorganic Chemistry*, **22**, 765–774.

Baldari,S. *et al.* (2019) Effects of copper chelation on BRAFV600E positive colon carcinoma cells. *Cancers*, **11**, 659.

Brooks,S.A. *et al.* (2016) miRNAs as common regulators of the transforming growth factor (TGF)-β pathway in the preeclamptic placenta and cadmium-treated trophoblasts: Links between the environment, the epigenome and preeclampsia. *Food and Chemical Toxicology*, **98**, 50–57.

Chan,N. *et al.* (2017) Influencing the Tumor Microenvironment: A Phase II Study of Copper Depletion Using Tetrathiomolybdate in Patients with Breast Cancer at High Risk for Recurrence and in Preclinical Models of Lung Metastases. *Clinical Cancer Research*, **23**, 666–676.

Chellan,P. and Sadler,P.J. (2015) The elements of life and medicines. *Philos Trans A Math Phys Eng Sci*, **373**, 20140182.

Choi,S. *et al.* (2018) Zinc deficiency and cellular oxidative stress: prognostic implications in cardiovascular diseases. *Acta Pharmacol Sin*, **39**, 1120–1132.

Denoyer,D. *et al.* (2015) Targeting copper in cancer therapy:‘Copper That Cancer’. *Metallomics*, **7**, 1459–1476.

Fatfat,M. *et al.* (2014) Copper chelation selectively kills colon cancer cells through redox cycling and generation of reactive oxygen species. *BMC cancer*, **14**, 1–12.

Finney,L. *et al.* (2009) Copper and angiogenesis: unravelling a relationship key to cancer progression. *Clin Exp Pharmacol Physiol*, **36**, 88–94.

Fouani,L. *et al.* (2017) Metals and metastasis: exploiting the role of metals in cancer metastasis to develop novel anti-metastatic agents. *Pharmacological Research*, **115**, 275–287.

Gandin,V. *et al.* (2012) A novel copper complex induces paraptosis in colon cancer cells via the activation of ER stress signalling. *Journal of cellular and molecular medicine*, **16**, 142–151.

Ge,E.J. *et al.* (2022) Connecting copper and cancer: from transition metal signalling to metalloplasia. *Nat Rev Cancer*, **22**, 102–113.

Gupte,A. and Mumper,R.J. (2009) Elevated copper and oxidative stress in cancer cells as a target for cancer treatment. *Cancer treatment reviews*, **35**, 32–46.

Jomova,K. *et al.* (2022) Essential metals in health and disease. *Chemico-Biological Interactions*, **367**, 110173.

Kozono,S. *et al.* (2018) Arsenic targets Pin1 and cooperates with retinoic acid to inhibit cancer-driving pathways and tumor-initiating cells. *Nat Commun*, **9**, 3069.

Liang,Y. *et al.* (2021) Rapamycin antagonizes cadmium-induced breast cancer cell proliferation and metastasis through directly modulating ACSS2. *Ecotoxicology and Environmental Safety*, **224**, 112626.

Liao,Y. *et al.* (2020) Inflammation mobilizes copper metabolism to promote colon tumorigenesis via an IL-17-STEAP4-XIAP axis. *Nat Commun*, **11**, 900.

Liu,Y.L. *et al.* (2021) Tetrathiomolybdate (TM)-associated copper depletion influences collagen remodeling and immune response in the pre-metastatic niche of breast cancer. *npj Breast Cancer*, **7**, 1–11.

Lopez,J. *et al.* (2019) Copper depletion as a therapeutic strategy in cancer. *Met. Ions Life Sci*, **19**, 303–330.

Marreiro,D. do N. *et al.* (2017) Zinc and Oxidative Stress: Current Mechanisms. *Antioxidants (Basel)*, **6**, 24.

Mehri,A. (2020) Trace Elements in Human Nutrition (II) – An Update. *Int J Prev Med*, **11**, 2.

Naji,S. *et al.* (2019) Cadmium induces migration of colon cancer cells: roles of reactive oxygen species, P38 and cyclooxygenase-2. *Cell Physiol Biochem*, **52**, 1517–1534.

Punshon,T. *et al.* (2019) Placental metal concentrations in relation to placental growth, efficiency and birth weight. *Environ Int*, **126**, 533–542.

Ramchandani,D. *et al.* (2021) Copper depletion modulates mitochondrial oxidative phosphorylation to impair triple negative breast cancer metastasis. *Nat Commun*, **12**, 7311.

Shi,H. *et al.* (2021) Cadmium induces epithelial–mesenchymal transition and migration of renal cancer cells by increasing PGE2 through a cAMP/PKA-COX2 dependent mechanism. *Ecotoxicology and Environmental Safety*, **207**, 111480.

Wu,F. *et al.* (2022) Bioinformatics analysis of key genes and potential mechanism in cadmium-induced breast cancer progression. *Environmental Science and Pollution Research*, **29**, 11883–11892.

Zhang,S. *et al.* (2015) Placental adaptations in growth restriction. *Nutrients*, **7**, 360–389.
